# Aluminium in the Arts and Manufactures

**Published:** 1888-12

**Authors:** 


ALUMINIUM IN THE ARTS AND MANUFACTURES.—Aluminium is destined to become before long of great importance in skilled art work and in manufactures, both in its pure state and as an alloy with other metals. It is as our readers possibly know, a product of clay, and it is estimated that in every cubic yard of clay there are at least eight hundred pounds of aluminium. But the process of extracting it is so costly that the pure metal can only be used in the manufacture of philosophical and other instruments where lightness is desired. It is in demand for theodolites, levels, compasses, sextants, for beams for chemical balances, opera and field glasses, certain kinds of surgical instruments, fine wire lace, suture wire, dental plates, &c. The demand is now about to be met by the manufacture of pure aluminium on a large scale by the aluminium company which has built works near Birmingham, England, for reducing the aluminium from the double chloride of aluminum and sodium at the rate of five hundred pounds of aluminium per day. As the production of the entire world has not heretofore exceeded fifty pounds per day, the new company can manufacture in five or six weeks as much as was formerly produced in a year, and it has reduced the selling price by cheapening the production of the metallic sodium from eleven dollars to five dollars per pound. But it is as an alloy in strengthening other metals that an extensive demand for the impure aluminium obtained by Cowles's electric process is springing up. Two works are now using this process. One of them is at Lockport, New York; the other at Milton, England, and both are reported to be running day and night. It is the extraordinary properties of the aluminium alloys which are rapidly bringing them not only into notice, but into use. Aluminium bronze is the strongest of cast metals, and when rolled the strongest of rolled metals. Aluminium brass is noted for its great strength, its high elastic limit and its cheapness. This cheapness arises from the fact that it contains an extremely small percentage of aluminium. There is hardly any metal with which it will not alloy, and it alloys with nothing that it does not improve. Its most remarkable peculiarity is the strength-giving property a small quantity of it imparts to all metals into which it enters as an alloy. Aluminium bronze is expected to play an important part in future wars, and also as an alloy for wrought and cast-steel guns, while repeated tests are said to show that one-tenth of one per cent. added to cast iron not only strengthens the casting, but solidfies it by preventing blow holes. Various metals have been used in the manufacture of propellers. Cast iron has been found too weak: steel is liable to corrode, the average life of a steel blade being only four years. In building the new steamships the government at first decided to use manganese bronze for the blades, at a cost of five hundred and sixty dollars per ton, but on its attention being drawn to aluminium brass a number of comparative tests were directed to be made, which resulted as follows:

| | *Mang. Bronze.* | *Al. Brass:* |
|---|---|---|
| Tensile strength per square inch, tons. | 29–31 | 34–50 |
| Transverse strength per square inch, cwts. | 28–52 | 75–85 |

The aluminium brass, costing about five hundred dollars per ton, was found to be of so much greater tensile and transverse strength that the government countermanded the order for manganese bronze and adopted aluminium brass in its stead.

